# Immune Responses in Pregnant Sows Induced by Recombinant *Lactobacillus johnsonii* Expressing the COE Protein of Porcine Epidemic Diarrhea Virus Provide Protection for Piglets against PEDV Infection

**DOI:** 10.3390/v14010007

**Published:** 2021-12-21

**Authors:** Dianzhong Zheng, Xiaona Wang, Ning Ju, Zhaorui Wang, Ling Sui, Li Wang, Xinyuan Qiao, Wen Cui, Yanping Jiang, Han Zhou, Yijing Li, Lijie Tang

**Affiliations:** 1 Heilongjiang Key Laboratory for Animal Disease Control and Pharmaceutical Development, College of Veterinary Medicine, Northeast Agricultural University, Harbin 150030, China; dianzhongzheng11@163.com (D.Z.); xiaonawang0319@163.com (X.W.); ju2645295669@163.com (N.J.); rui10151108@163.com (Z.W.); Isseven111@163.com (L.S.); wanglicau@163.com (L.W.); qiaoxinyuan@126.com (X.Q.); cuiwen_200@163.com (W.C.); jiangyanping2017@126.com (Y.J.); zhouhan9659@163.com (H.Z.); 2 Northeastern Science Inspection Station, China Ministry of Agriculture Key Laboratory of Animal Pathogen Biology, Harbin 150030, China

**Keywords:** *Lactobacillus johnsonii* (*L. johnsonii*), COE antigen, immune protection, pregnant sow, oral immunization, PEDV

## Abstract

Porcine epidemic diarrhea (PED) induced by porcine epidemic diarrhea virus (PEDV) is an intestinal infectious disease in pigs that causes serious economic losses to the pig industry. To develop an effective oral vaccine against PEDV infection, we used a swine-origin *Lactobacillus johnsonii* (*L. johnsonii*) as an antigen delivery carrier. A recombinant strain pPG-T7g10-COE/*L. johnsonii* (*L. johnsonii-COE*) expressing COE protein (a neutralizing epitope of the viral spike protein) was generated. The immunomodulatory effect on dendritic cell in vitro and immunogenicity in pregnant sows was evaluated following oral administration. *L. johnsonii-COE* could activate monocyte-derived dendritic cell (MoDC) maturation and triggered cell immune responses. After oral vaccination with *L. johnsonii-COE*, levels of anti-PEDV-specific serum IgG, IgA, and IgM antibodies as well as mucosal secretory immunoglobulin A (SIgA) antibody were induced in pregnant sows. High levels of PEDV-specific SIgA and IgG antibodies were detected in the maternal milk, which provide effective protection for the piglets against PEDV infection. In summary, oral *L. johnsonii-COE* was able to efficiently activate anti-PEDV humoral and cellular immune responses, demonstrating potential as a vaccine for use in sows to provide protection of their piglets against PEDV.

## 1. Introduction

Porcine epidemic diarrhea virus (PEDV) infection can cause anorexia, vomiting, and watery diarrhea, and ultimately lead to piglet death. PEDV encodes four structural proteins: spike (S), matrix (M), nucleocapsid (N), and envelope (E) proteins. The S protein plays a necessary function in virus invasion of the host cells, in addition, it can induce the production of anti-PEDV specific antibodies [1]. The S protein of PEDV can be divided into four neutralizing epitope domains: COE (499–638 aa), SS2 (748–755 aa), SS6 (764–771 aa), and 2C10 (1368–1374 aa) [2]. The protective effect of the orally administered recombinant *Lactobacillus* expressing the COE gene against PEDV infection in a mouse model had been investigated [3], and the results showed that recombinant *Lactobacillus* induced significant levels of anti-PEDV specific IgG and SIgA antibody, implying a potential vaccine strategy against PEDV infection.

Naturally, PEDV infection often invades the host via the intestinal mucosal surface. It has been reported sow IgA-plasmablasts and T cells in the intestine were activated by oral immunization with recombinant *Lactobacillus acidophilus* expressing S protein of PEDV to induce protective mucosal immunity [2]. In addition, IgA-plasmablasts can migrate to the mammary tissue to produce secretory immunoglobulin A (SIgA) antibody in milk [4]. The immune system of newborn piglets is not fully developed. As a result, traditional vaccines do not effectively produce protection due to the inability to produce immune response in time [2]. Therefore, an ideal method is to produce maternal antibodies that can be passively transferred to neonatal pigs via colostrum in immunized pregnant sows [5]. Specific SIgA and IgG antibodies produced by pregnant sows immunized with vaccines can be delivered to newborn piglets and provide immune protection against viral infections [2,6,7]. 

The oral administration route is more convenient than the traditional injection route. *Lactobacilli* have a clear advantage as an oral vaccine carrier including safety, adjuvant properties, mucosal adhesive properties, and low intrinsic immunogenicity [8,9,10]. At present, *Lactobacilli* have been successfully constructed to express antigens to induce the production of specific IgG and SIgA antibodies in animals by oral immunization [11,12,13].

Dendritic cells (DCs) are an antigen-presenting cells of the mammalian immune system, which have a pivotal role in activating the intestinal immune response and can promote the proliferation and differentiation of naïve T cells. Upon detection of the exogenous antigen, DCs secrete pro-inflammatory cytokines to regulate Th1 immune responses through specific molecular pattern recognition receptors [14].

The COE antigenic domains of PEDV can induce specific anti-PEDV neutralizing antibodies, which have been widely investigated as potential candidates for the development of vaccines against PEDV [3,13]. In the present study we constructed a recombinant *L. johnsonii* of swine origin that expressed PEDV COE protein. Following oral delivery to pregnant sows, effect on immunogenicity was evaluated. The protective effect against PEDV transferred from immunized sows to their suckling piglets was assessed.

## 2. Materials and Methods

### 2.1. Bacterium, Plasmid and Virus

*Lactobacillus johnsonii* (GenBank Accession No. MH492312) isolated from the intestinal mucus of pig by our laboratory. The constitutive expression plasmid pPG-T7g10-COE was constructed in our laboratory [15]. PEDV strain HLJ-2012 was isolated from the intestines of piglets with severe diarrhea in Heilongjiang, China, and was maintained in our laboratory [15].

### 2.2. Construction of Recombinant Lactobacillus johnsonii

The constitutive expression plasmid pPG-T7g10-COE was transformed into *L. johnsonii* competent cells by electroporation under 2000 V/cm; then, the treated *L. johnsonii* was cultured in MRS broth medium contain 0.3 M sucrose, and the recombinant strain *L. johnsonii-COE* was obtained on MRS agar medium containing 10 μg/mL chloromycetin as previously described [3]. 

### 2.3. Protein Expression by Recombinant Lactobacillus johnsonii

The recombinant *L. johnsonii-COE* was cultivated statically in MRS medium containing 10 μg mL^−1^ chloromycetin at 37 °C. The bacteria were harvested by centrifugation at 12,000× *g* for 1 min, and washed with sterile phosphate-buffered saline (PBS) twice. The bacteria were lysed by ultrasound treatment, pelleted by centrifugation, and the sediment was analyzed by western blotting as previously described [15], using the rabbit anti-COE monoclonal antibody (prepared in our laboratory) and horseradish peroxidase (HRP)-conjugated goat anti-rabbit IgG antibody (Thermo Fisher, San Jose, CA, USA) as primary and secondary antibodies, respectively.

### 2.4. Isolation and Culture of Porcine Monocyte-Derived Dendritic Cells (MoDCs)

MoDCs were obtained as previously described [16]. In brief, monocytes were generated from the peripheral blood of piglets. The monocytes were differentiated into DCs using 20 ng mL^−1^ GM-CSF (R&D systems, Minneapolis, MN, USA) and 20 ng mL^−1^ IL-4 (R&D systems, Minneapolis, MN, USA) for six days. The morphology of the MoDCs was observed using light microscopy. 

### 2.5. Scanning Electron Microscopy (SEM) Observation

The morphology of MoDCs and *L. johnsonii-COE* adheres to MoDCs were investigated by SEM as previously described [16]. In brief, *L. johnsonii-COE* (10^6^ CFU) were co-incubated with MoDCs for 90 min. Subsequently, MoDCs were washed with PBS. The MoDCs were fixed with 2% glutaraldehyde for 3 h at 4 °C and washed thrice with 0.1 M PBS (pH 7.2). The MoDCs were dehydrated in 50%, 70%, 90%, and 100% ethanol solutions, followed by treatment with a mixture of ethanol, tertiary butanol, and pure tertiary butanol. Samples were covered with gold after freeze-drying and analyzed under an SU8200 SEM (Hitachi, Tokyo, Japan).

### 2.6. Detection of MoDCs Surface Markers

The immature MoDCs were activated by *L. johnsonii-COE* (10^6^ CFU) for 12 h, and LPS (200 ng mL^−1^, Sigma, Ronkonkoma, NY, USA), *L. johnsonii*, and unstimulated group as control. MoDCs were stained with PE-conjugated anti-CD172a monoclonal antibodies (Southern Biotech, Birmingham, AL, USA), PE-conjugated anti-CD80 monoclonal antibodies (Thermo Fisher Scientific, USA), and FITC-conjugated anti-MHCII (DR+DP) monoclonal antibodies (Abcam, Cambridge, Cambridgeshire, UK) and the phenotype was analyzed using flow cytometry.

### 2.7. Relative Expression Analysis of Cytokines

*L. johnsonii-COE* (10^6^ CFU) stimulated MoDCs for 12 h, and *L. johnsonii* or LPS (200 ng mL^−1^) as control. Afterward, the total cellular RNA of MoDCs were extracted, and the cDNA were generated by RNA reverse transcriptase and oligo (dT) primer (TaKaRa, Dalian, China) as previously described [16]. The mRNA levels of Th1-type cytokines IL-12p40 and Th2-type cytokines IL-10, and chemokine CCL20 were determined by qPCR. β-actin was used as housekeeping gene. The expression of objective gene was calculated using the 2^−ΔΔCt^ method [17]. Primers were listed in Table 1.

### 2.8. L. johnsonii-COE Activated MoDCs Induced CD4+ T Cell Proliferation and Differentiation

Allogeneic CD4^+^ T cells were generated as previously described [16]. First, MoDCs were stimulated with *L. johnsonii-COE* (10^6^ CFU) for 12 h, LPS (200 ng mL^−1^) and *L. johnsonii* as control. Subsequently, the MoDCs were co-cultivated with allogeneic CD4^+^ T cells for 72 h (The ratio of dendritic cells to T cells is 1:10). The control group including CD4^+^ T cells, MoDCs, and cell medium wells. After 72 h, CCK-8 (10 uL well^−1^; Sigma) (a kind of orange dye by the interaction with cell mitochondria, which is directly proportional to the number of living cells) was added into each sample wells, and the OD450 of each well was measured using a microplate reader after further cultured 5 h. The SI (stimulation index) was calculated using the following formula: SI = (OD (MoDC+T) 450 nm − OD (MoDC) 450 nm)/(OD (T) 450 nm − OD (medium) 450 nm).

The culture supernatants were collected from MoDCs treated by *L. johnsonii-COE*, LPS, and *L. johnsonii* co-cultured with CD4^+^ T cells as described above. CD4^+^ T cells co-cultured with unstimulated MoDCs as control. The yield of cytokines IL-12p40, IL-10, and IL-17 were investigated by enzyme-linked immunosorbent assay (ELISA) kits (Cusabio, Wuhan, China).

### 2.9. Oral Immunization of Pregnant Sows

**Figure 1:**
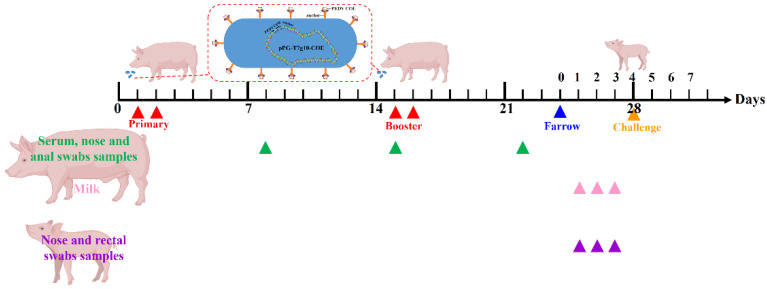
Immunization protocol and sampling schedule.

Animal experiments were carried out in accordance with the recommendations in the institutional and national guidelines for animal care and use. The protocol was approved by the Committee on the Ethics of Animal Experiments of Northeast Agricultural University, Harbin, China (2016NEFU-315, 13 April 2017). Six large white sows at 90 days of gestation were obtained from the experimental animal base of Northeast Agricultural University (Harbin, Heilongjiang Province, China). All sows were determined to be antigen-negative for PEDV by PCR and ELISA, respectively. Six pregnant sows were randomly divided into two groups (three sows per group) and were fed in similar conditions in different hogcotes. The vaccine group of the sows were orally vaccinated (mixed with feed) with 100 mL of recombinant *L. johnsonii-COE* (ca. 1 × 10^11^ CFU). The control group of pregnant sows was fed normally. As shown in Figure 1, the immunization project was carried out on two consecutive days (Days 1 and 2), and booster immunization was given on Days 15 and 16. At 8, 15, and 22 days after primary immunization, and serum samples were collected. Rectal swab samples were taken with sterile swabs inserted 40–50 mm into rectum and rotated against the bowel wall, and rectal swabs were placed and thoroughly mixed in 2 mL Eppendorf tubes prefilled with 1 mL sterile PBS for 12 h at 4 °C, and after centrifuge at 12,000 rpm at 4 °C for 1 min, and supernatants were stored at −80 °C until use). The nasal swab samples collection is the same as the description of rectal swab samples collection [18,19]. Farrowing was induced nine days after the last immunization. Colostrum samples were harvested at 24, 48, and 72 h after delivery. All samples were stored at −80 °C until use.

### 2.10. Analysis of Antibody Levels by ELISA

The levels of anti-PEDV IgG, IgA, and IgM antibodies in the serum, SIgA, and IgG antibodies in the colostrum, nasal mucosa, and rectal mucosa were analyzed by ELISA. The ELISA methods were referenced to previous study [12]. In brief, PEDV HLJ-2012 (200 TCID50) was used to coat a 96-well polystyrene plate at 4 °C overnight. After washing, the plate was blocked with 5% skim milk at 37 °C for 2 h. After washing, the wells were incubated with sera samples (diluted at 1:10), colostrum samples (diluted at 1:10), and nose and rectal swab samples at 37 °C for 1 h. After washing, the wells were incubated with goat anti-pig IgG or IgA antibody (HRP-conjugated, diluted at 1:5000) (Abcam, Cambridge, Cambridgeshire, UK) at 37 °C for 1 h followed by washing. Absorbance was measured at OD450 nm by color change of o-phenylenediamine dihydrochloride (Sigma, Ronkonkoma, NY, USA). In addition, the levels of cytokines in serum, including IL-2, IL-4, IL-6, IL-12, IL-17, and IFN-γ, were measured using ELISA kits (Meimian, China).

### 2.11. Analysis of Antibody Levels in Piglets

Neonatal piglets were allowed to suckle from sows for 3 days, during which piglet nasal and rectal swab were harvested every day. On the fourth day, serum samples were harvested from the piglets. The levels of SIgA and IgG antibodies in the nasal mucosa and rectal mucosa and IgA and IgG antibodies in serum were analyzed by ELISA.

### 2.12. Challenge, PEDV Fecal Shedding Analysis and Clinical Evaluation

Neonatal piglets were allowed to suckle from sows for 3 days, and six piglets were randomly selected from each group, afterward, piglets were orally challenged with 2 mL containing 10^4^ TCID50 of PEDV tissue virulent virus (Figure 1). During the challenge period, the number of piglets with diarrhea was recorded per 12 h. At the 60th hour, fecal samples of piglets were collected and the RNA levels of PEDV was detected by RT-qPCR assay, the primer sequence of qPCR was shown in Table S1. 

### 2.13. Histopathological Analysis

All piglets were euthanized after 60 h of challenge and ileum tissue were collected to evaluate histological changes. In brief, intestinal tissues segments were fixed by 4% paraformaldehyde, dehydrated, embedded in paraffin, sectioned, stained with haematoxylin and eosin (H&E) and then examined for pathological changes by Olympus BX53M microscope as previously described [1].

### 2.14. Immunohistochemistry

PEDV was detected in ileum tissues from piglets of control group and *L. johnsonii-COE* group. In brief, tissues segments were fixed by 4% paraformaldehyde, paraffin-embedded, sectioned, dewaxed and blocked. The sections were incubated with mouse anti-pig N protein of PEDV polyclonal antibodies (prepared by our laboratory and diluted 1:10). HRP-labeled anti-mouse secondary antibody was used for incubating the sections. Following staining with diaminobenzidine (DAB), counterstaining with hematoxylin, dehydrated, and analyzed as previously described [20].

### 2.15. Statistical Analysis

Significant differences in the nasal mucosa, rectum mucosa, serum, and colostrum antibody levels of different groups, cytokine expression, T cells proliferation, and surface markers in MoDCs analysis were compared using a one-way analysis of variance (ANOVA) general linear model followed by Duncan’s multiple range test. All data are representative of three independent experiments, and analyzed by the GraphPad Prism 7.0. The results are exhibited as means ± standard deviations (SD). * *p* < 0.05 represent statistically significant, ** *p* < 0.01 represent highly significant.

## 3. Results

### 3.1. Analysis of COE Expression in L. johnsonii

**Figure 2:**
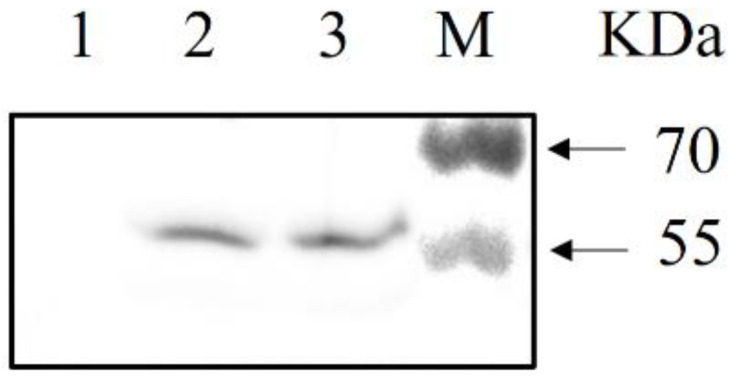
Western blotting for the detection of COE protein. A protein band of approximately 59 kDa corresponding to COE was detected using rabbit anti-COE monoclonal antibody (Lanes 2 and 3). *Lactobacillus johnsonii* as the negative control (Lane 1). The band size was consistent with the expected size.

The protein expression of the recombinant strain *L. johnsonii-COE* was detected by western blotting. The COE fusion protein with a size of approximately 59 kDa could be effectively recognized by the rabbit anti-COE monoclonal antibody, and no band was present in wild type *L. johnsonii* (Figure 2).

### 3.2. Morphological Observation of Porcine MoDCs and L. johnsonii-COE Adherence to MoDCs

**Figure 3:**
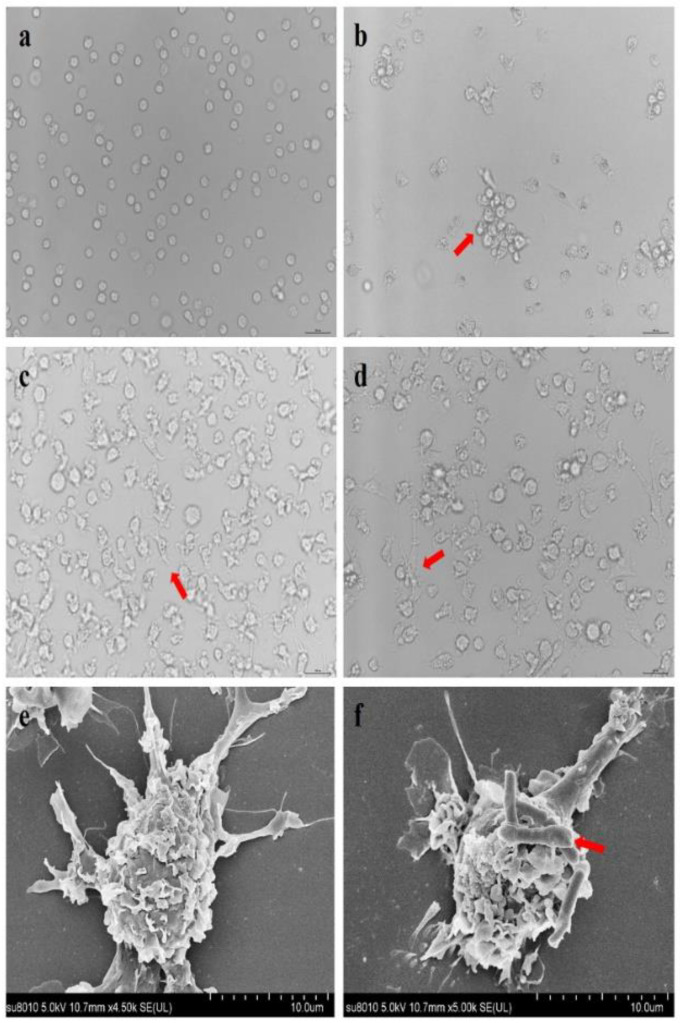
Porcine MoDCs formation at different cultured period and adhesion of *L. johnsonii-COE* to MoDCs. (**a**–**d**) The images were observed by light microscopy*,* and scale is 100 um*;* (**a**) CD172a^+^ monocytes; (**b**) monocytes on 2 days; (**c**) monocytes on 4 days; (**d**) MoDCs on 6 days; the red arrows in (**b**–**d**) present dendritic morphology. (**e**) MoDCs seen by SEM; (**f**) some *L. johnsonii-COE* adhere to MoDCs under SEM, the red arrows present *L. johnsonii-COE*.

As shown in Figure 3a, the CD172a monocytes were generated from the PBMCs and cultured in RPMI-1640 medium containing GM-CSF and IL-4. After two days, small dendritic structures and cell aggregation were observed (Figure 3b). The cells grew a distinct dendritic structure until four days (Figure 3c). After six days, most of the cells differentiate into dendritic cells (Figure 3d). Porcine MoDCs were observed using SEM (Figure 3e). Some *L. johnsonii-COE* adhered to the MoDCs surface (Figure 3f).

### 3.3. L. johnsonii-COE Activated the Maturation of the MoDCs by Flow Cytometry

**Figure 4:**
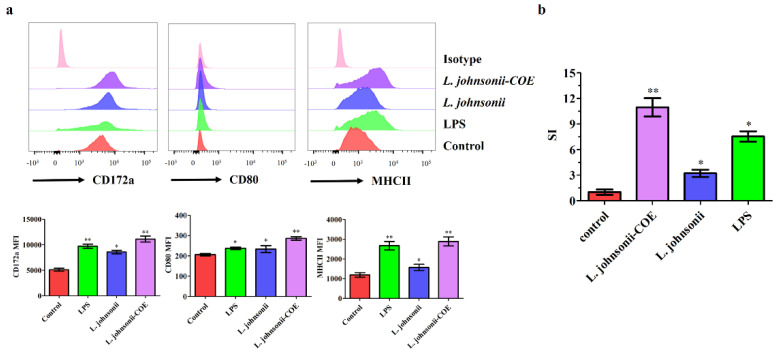
Analysis of surface markers and CD4^+^ T cells proliferation in MoDCs stimulated by *L. johnsonii–COE*, LPS (200 ng mL^−1^), and *L. johnsonii*. (**a**) The expression of CD172a, CD80, and MHCII on MoDCs were detected via flow cytometry, and the mean fluorescence intensity (MFI) values of represent three molecules are exhibited as mean ± SD values; (**b**) analysis of proliferation of differently treated MoDCs induced CD4^+^ T cells. * (*p* < 0.05) and ** (*p* < 0.01).

Molecular markers on the surface of DC such as CD172a, MHCII and CD80 participate in the antigen presentation and stimulating T cell activation [16]. Upregulation of the molecular markers on the cell surface means maturation of MoDCs [21]. To study the role of *L. johnsonii-COE* activated MoDCs maturation, *L. johnsonii-COE* stimulated MoDCs for 12 h. As shown in Figure 4a, the expression of CD172a, CD80, and MHCII markedly increased in *L. johnsonii-COE* compared to that in *L. johnsonii* and the control. The results indicated that *L. johnsonii-COE* induced MoDCs maturation.

### 3.4. MoDCs Activated by L. johnsonii-COE Promoted CD4^+^ T Cell Proliferation

Porcine CD4^+^ T cells were isolated by MACS screening as previously described [16]. To determine if *L. johnsonii-COE* stimulated MoDCs induce CD4+ T cells proliferation, *MoDCs* was co-cultured with *L. johnsonii-COE* for 12 h. Proliferation of T cells was measured by CCK-8. As shown in Figure 4b, the stimulation index (SI) significantly increased in the *L. johnsonii-COE* group compared with others, suggesting that *L. johnsonii-COE* stimulated MoDCs could promote the proliferation of CD4*^+^* T cells.

### 3.5. L. johnsonii-COE Activated MoDCs Expressed Cytokines

**Figure 5:**
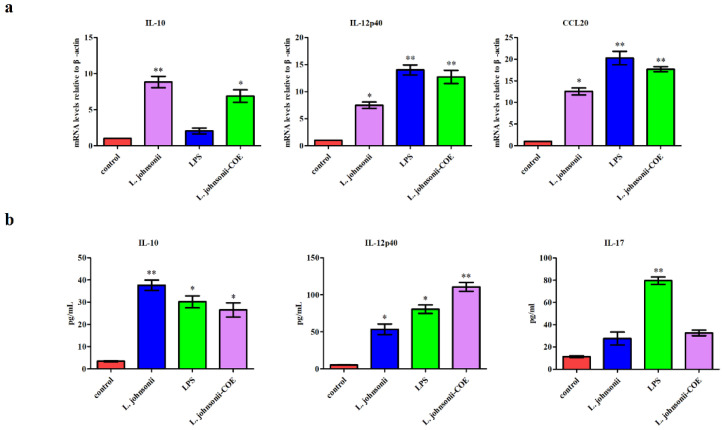
The expression of cytokines in MoDCs primed by *L. johnsonii-COE*, LPS, and *L. johnsonii* and analysis of effector T cell differentiation. (**a**) Real-time PCR measured the transcriptional of IL-12p40, IL-10, and CCL20 in MoDCs. (**b**) Different treated MoDCs co-cultivated with allogenic CD4^+^ T cells for 72 h, medium was collected and ELISA analysis of the cytokines secretion of IL-12p40, IL-10, and IL-17. * (*p* < 0.05) and ** (*p* < 0.01).

Cytokines play a key role in T cells differentiation [16]. Expression of cytokines is an indicator of the ability of *L. johnsonii-COE* to modulate the immune function of the DCs. We measured the mRNA levels of some cytokines, including Th2-type cytokines IL-10, Th1-type cytokines IL-12p40, and the chemokine CCL20. The result showed that DCs activated by *L. johnsonii-COE* resulted in significantly increased mRNA expression of IL-12p40 and CCL20 than IL-10 (Figure 5a), indicating that *L. johnsonii-COE* induced cytokines typically involved in a Th-1 type cellular immune response. 

### 3.6. MoDCs Activated by L. johnsonii-COE Have Ability to Drive CD4^+^ T Cell toward Th1 Subset Polarization

To investigate the ability of *L. johnsonii-COE* treated MoDCs to drive CD4*^+^* T cells differentiation, activated MoDCs were co-cultured with CD4*^+^* T cell. The supernatant was obtained from DC-T cell co-culture. We measured some cytokines related to different Th cell subsets (Figure 5b). The result showed that MoDCs primed by *L. johnsonii-COE* resulted in significantly increased release of the Th1-type cytokine IL-12p40 rather than IL-10 and IL-17. Further, the persistence of IL-12 produced by DCs is necessary to maintain Th1 type responses. The results mean that the *L. johnsonii-COE* can effectively activate MoDCs and mediate Th1-type cellular responses.

### 3.7. PEDV-Specific Antibody Levels in Sows Induced by L. johnsonii-COE

**Figure 6:**
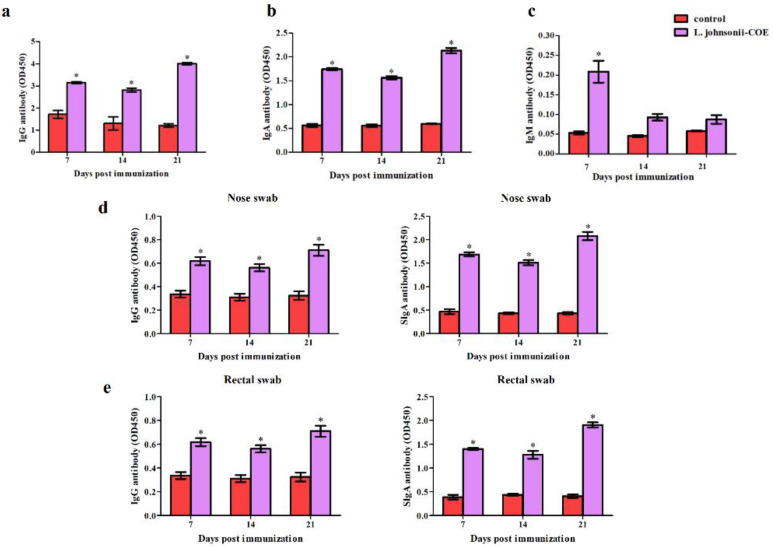
The IgG, IgA, SIgA, and IgM antibody levels of the immunized sow. (**a**–**c**) Serum anti-PEDV IgG (**a**), IgA (**b**), IgM (**c**) antibody levels; (**d**) nasal mucosa anti-PEDV IgG and SIgA antibody levels; (**e**) rectal mucosa anti-PEDV IgG and SIgA antibody levels. * (*p* < 0.05).

To investigate the immunogenicity of the *L. johnsonii-COE*, six pregnant sows were used for immunization, and the production of specific antibodies were detected using ELISA. As shown in Figure 6a–c, *L. johnsonii-COE* significantly stimulated the specific serum IgG and IgA antibodies production after the first and second immunizations contrasted with the control group. A high level of specific IgM antibody was also detected after primary immunization in the *L. johnsonii-COE* group. Moreover, as shown in Figure 6d,e, significant levels of SIgA and IgG antibodies in the rectal mucosa and nasal mucosa were induced by recombinant *L. johnsonii-COE* on Day 7 post the primary immunization. Furthermore, the expression of these antibodies obviously increased after the second immunization.

### 3.8. Cytokines Detection in the Serum of Pregnant Sows

**Figure 7:**
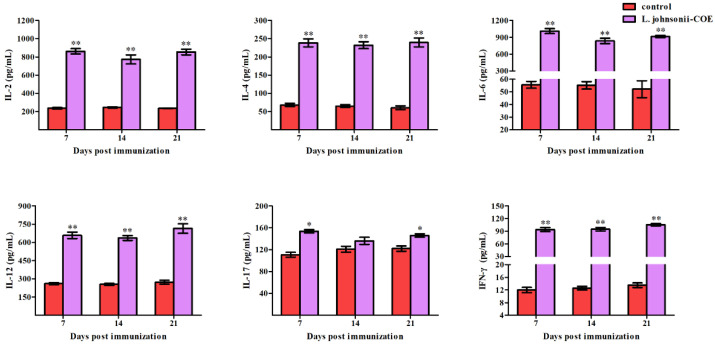
Levels of the cytokines in immunized sow. Cytokines including IL-2, IL-4, IL-6, IL-12, IL-17, and IFN-γ levels in serum of sow immunized with *L. johnsonii-COE*, and control group with fed normally were analyzed by ELISA. * (*p* < 0.05) and ** (*p* < 0.01).

The cytokine levels in the serum of pregnant sows in each group are shown in Figure 7. Notably, higher concentrations of IL-2, IL-6, and IL-12 were measured in the *L. johnsonii-COE* group. In addition, *L. johnsonii-COE* significantly induced the expression of IL-4, IL-17, and IFN-γ than the control group. 

### 3.9. Antibody Levels in the Colostrum

**Figure 8:**
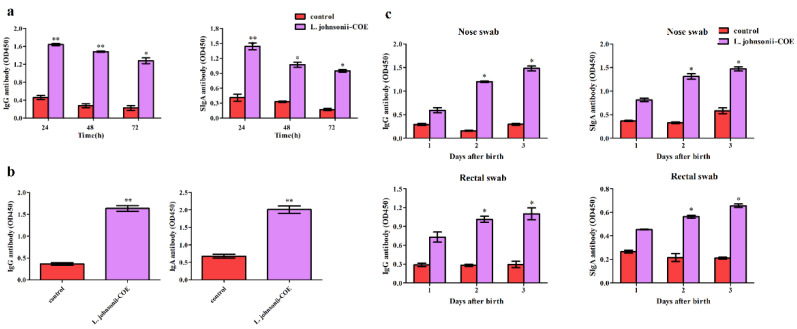
Levels of the antibodies in colostrum and piglets. (**a**) Colostrum anti-PEDV SIgA and IgG antibody levels of the sow groups immunized with *L. johnsonii-COE*; (**b**) serum anti-PEDV SIgA and IgG antibody levels of the 4-day-old piglets; (**c**) nasal mucosa and rectal mucosa anti-PEDV SIgA and IgG antibody levels of the piglets at 1 day, 2 day, and 3 days of age. * (*p* < 0.05) and ** (*p* < 0.01).

Neonatal piglets were termly suckled by the immunized sows, and the colostrum antibodies can transfer passive immunity to the neonatal piglets [2]. In this study, colostrum samples were obtained from the sows at 24, 48, and 72 h after farrowing for the presence of anti-PEDV SIgA and IgG antibodies. As shown in Figure 8a, the production of specific anti-PEDV SIgA and IgG antibodies in the colostrum of the *L. johnsonii-COE* group were significantly higher than those in the control group. Furthermore, quantity of antibodies in colostrum declined with time.

### 3.10. Detection of Specific Anti-PEDV SIgA and IgG Antibodies in Piglets

To determine the level of specific antibodies in piglets, as a result of suckling, IgA and IgG antibodies in the sera of four-day-old piglets and nasal mucosa and rectal mucosa at one, two, and three days of age were analyzed using ELISA. The levels of IgA and IgG antibodies were markedly increased in the piglet serum of the recombinant *L. johnsonii-COE* group than in the control group (Figure 8b). Furthermore, compared to the control group, SIgA and IgG levels in piglet mucosal regions, such as the nasal mucosa and the rectal mucosa, showed an increasing trend in the *L. johnsonii-COE* group (Figure 8c).

### 3.11. The Detection of Fecal Viral Shedding in Piglets and Clinical Symptoms Evaluation

**Figure 9:**
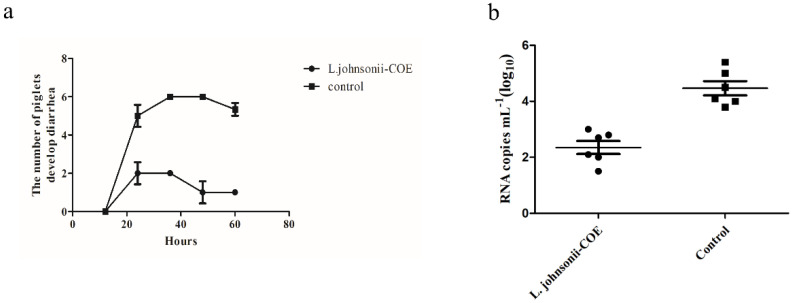
Detection of the levels of virus RNA in feces of the piglets and immune protective efficiency after challenging. (**a**) The quantity of piglets with diarrhea in the vaccinated *L. johnsonii–COE* group and normal breeding group after PEDV challenging. (**b**) RT–qPCR analyzed PEDV excretion in piglets.

All the piglets were determined to be negative for PEDV. After virulent PEDV challenge, diarrhea developed in the control group at 24 h, peaking at 36 h and 48 h (when 6 piglets had appeared diarrhea). However, the *L. johnsonii-COE* immunized group showed a reduced number of diarrhea piglets (Figure 9a). Fecal samples were obtained from all piglets and the copy number of the PEDV RNA in feces was evaluated. The result showed that *L. johnsonii-COE* groups had lower copy numbers than the control groups at 60 h (Figure 9b).

### 3.12. Histopathological Observation and Immunohistochemistry Analysis

**Figure 10:**
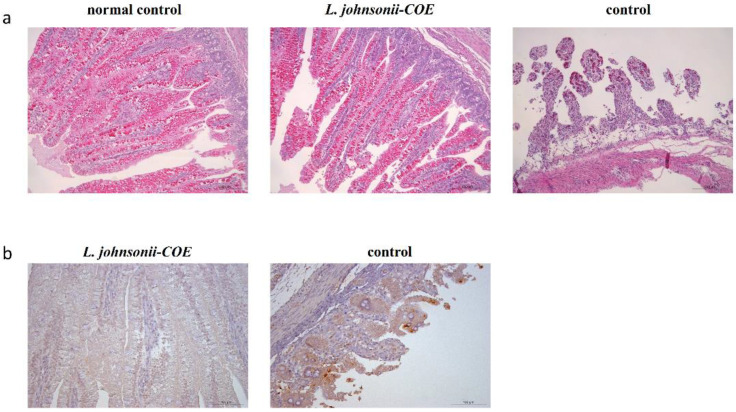
Histopathological observation (scale is 200 um) (**a**) and the detection of virus in the ileum of the piglets by immunohistochemistry (scale is 100 um) (**b**) at the 60th hour post-challenge with PEDV.

Pathological examination showed that compared to normal control group (without being challenged), there were no abnormal histopathological changes observed in the ileum of the piglets in the *L. johnsonii-COE* group. However, there were significant histopathological changes in the ileum of the piglets in the control group, including villous blunting and atrophy, loss of intestinal crypts, complete loss of epithelial cells, and complete loss of villous architecture (Figure 10a). Subsequently, we detected the virus in the intestinal mucosa of the piglets in each group by immunohistochemical assay, as shown in Figure 10b, there were a large number of viruses detected in the ileum of the piglets in control group, while almost no virus was detected in *L. johnsonii-COE* group.

## 4. Discussion

PEDV is an important viral pathogen in pig, causing huge losses to the pig industry worldwide [22]. Currently, prevention and control of PEDV transmission mainly depend on vaccination. However, a main defect of conventional vaccines is the animals have to be immunized many times to produce enough antibody levels, and this may be cause stress responses in piglets. In addition, invasion of PEDV usually starts at intestinal mucosal surfaces [23]. Therefore, inducing SIgA-based protective intestinal mucosal immune response through the oral route could effectively against PEDV infection. 

*Lactobacillus* strains are ideal oral vaccines delivery carriers because of their high safety, strong adhesion to the intestinal mucosa, and capacity to express heterologous proteins [11,12,24]. In previous study, recombinant *Lactobacillus casei* (*L. casei*) expressed a microfold cell-targeting peptide fused with PEDV COE antigen induced high levels of anti-PEDV IgG and SIgA antibody in in the mouse model [15]. However, the study was only applied to mouse models and not pigs, and *Lactobacillus casei* was not isolated from pigs. Therefore, using a swine-origin genetically engineered *Lactobacillus* expressing PEDV COE antigen was used to evaluate its immune effect in pigs via oral immunization, which may be more proof that recombinant *Lactobacillus* expressing PEDV COE antigen as a promising oral vaccine candidate for PEDV. In this study, *Lactobacillus johnsonii* isolated from the intestinal mucus of pig by our laboratory. Furthermore, we constructed a recombinant *L. johnsonii* that expressed PEDV COE protein. The recombinant *L. johnsonii* was evaluated in terms of immunogenicity including induction of systemic and mucosal PEDV specific antibodies in vivo and the ability of the bacteria to induce monocyte-derived dendritic cell maturation, cytokine secretion and T cell activation and proliferation in vitro.

In general, *lactobacilli* can adhere to the surface of intestinal mucosa, contact with the DCs in the lamina propria of mucosa, and activate DCs to induce immune responses. Our results showed that *L. johnsonii-COE* have the ability to adhere to MoDCs, indicating the basis of MoDCs activation. As an oral vaccine, the capability of activating DC maturation and mediating T cell proliferation and differentiation is indispensable to activate the cell immune response [25]. In this study, *L. johnsonii-COE* stimulated MoDCs maturation and significantly increased the expression of surface molecules in MoDCs, such as CD172a, CD80, and MHCII, implying that *L. johnsonii-COE* promoted the maturation of porcine MoDCs. Besides, *L. johnsonii-COE* stimulated MoDCs promoted CD4^+^ T cell proliferation. 

In addition to the maturation state, cytokines produced by DCs are necessary for inducing effector T cells production [26]. In addition to costimulatory molecules that can activate T cells through the antigen presentation, cytokines provide another pathway to activate naïve T cells proliferation and differentiation [26]. It is well known that IL-12p40 is Th1-associated cytokines, IL-10 is Th2-associated cytokines, and CCL20 is chemokine, they are related to adaptive immune response [27,28,29]. In this study, *L. johnsonii-COE* increased MoDCs-induced Th1-associated cytokines IL-12p40 and chemokine CCL20 expression compared with IL-10. IL-10 plays a role in Th2-associated cell differentiation [26,28]. Interestingly, MoDCs activated by *L. johnsonii-COE* modestly up-regulated the expression of the anti-inflammatory cytokine IL-10, which may be related to the ability of *Lactobacillus* to control inflammation [30]. High levels of IL-12p40, but not of IL-17 and IL-10, were monitored in the supernatant of MoDCs stimulated by *L. johnsonii-COE* co-cultivate with CD4^+^ T cells, representing that MoDCs treated by *L. johnsonii-COE* induce Th1-associated immunity, and the latter is important in protecting the host from obligate intracellular pathogens [31]. 

In general, PEDV caused severe clinical symptoms in neonatal pig before the formation of active immunity; therefore, compared to directly immunizing piglets, using the approach where in the neonates are protected by passive lactogenic immunity may serve to be more feasible [5]. The passive immunity is relied on the supplementation of maternal-derived immune components through mammary secretions, such as colostrum [32]. Therefore, immunizing sows with oral vaccine during gestation is an attractive approach to induce intestinal mucosal immune responses. *Lactobacillus* expressed antigen entered the intestine of sows by oral administration and continue to stimulate dendritic cells in the intestinal mucosa. Dendritic cells can not only activate lymphocytes to produce specific antibodies, but also induce more IgA-plasmablasts and T cells in the intestine. In addition to locating intestinal tracts, some IgA-plasmablasts and T cells migrate to the mammary tissue, producing SIgA and cytokines in milk [2,33]. Subsequently, SIgA and cytokines in milk were delivered to neonatal pig via mammary secretions. It is important to efficiently activate both mucosal and systemic immune responses for oral vaccine. Therefore, the immunogenicity of the *L. johnsonii-COE* was investigated in sows via oral administration. The SIgA antibody is beneficial to host against virus invasion through the mucosa. In this study, a higher level of anti-PEDV SIgA antibody was induced by the recombinant strain *L. johnsonii-COE* in the nasal mucosa and rectal mucosa of the sow. IgG, IgM, and IgA antibodies in the serum contributed to the immune protection via suppressing the spread of the virus in animals. Our results found that remarkable levels of anti-PEDV IgG, IgM, and IgA antibodies were induced in the serum of the sow. These antibodies may provide specific protection against PEDV infection. The levels of cytokines, including those of IFN-γ (related to the antiviral effect), IL-2, IL-6 (related to immune activation and immune cell proliferation and differentiation), IL-4 (related to humoral immunity), IL-12 (related to Th1 type cellular immune response), and IL-17 (related to Th17 type cellular immune response) in the serum of sows were detected [31,34]. The results showed that the high levels of IL-2, IL-4, IL-6, IL-12, and IFN-γ increased after immunization, indicating that the cellular and humoral immune responses could be activated in sows by *L. johnsonii-COE* via oral vaccination. Therefore, we determined that oral vaccination with strain *L. johnsonii-COE* delivering PEDV-protective antigen COE protein be able to effectively activate anti-PEDV mucosal and humoral immune responses. In previous study, the similar effects of COE protein have been demonstrated in mouse models [3]. However, it is unknown whether maternal antibodies can be delivered to newborn piglets. Therefore, we investigated the production of SIgA and IgG antibodies in the colostrum of sows orally vaccinated with *L. johnsonii-COE* and in newborn piglets. The results showed high levels of SIgA and IgG antibodies in the colostrum of vaccinated sows, as well as in the serum, nasal mucosa, and rectal mucosa of the piglets born to them. Thus, SIgA and IgG antibodies were delivered to the piglets via the colostrum.

In order to evaluate the immune protection of the strain *L. johnsonii-COE* for the passively immunized piglets against PEDV infection, we challenged piglets with PEDV tissue virulent virus. *L. johnsonii-COE* showed preventive effect on piglet diarrhea and virus infection. Histopathological observation revealed that significant histopathological changes, including disruption of the intestinal structural integrity, shortening of the villi, and largely necrotic and exfoliated of mucosal epithelial cells, in the intestine of the piglets which control group after PEDV challenge, while there were no abnormal histopathological changes observed in the intestines of the piglets in *L. johnsonii-COE* group. Moreover, immunohistochemistry assay and RT-qPCR showed that the virus could be gradually cleared from the intestine of the piglets in *L. johnsonii-COE* group, but not in the piglets of control group. Here, our results clearly demonstrated that the oral recombinant *L. johnsonii-COE* could provide effective immune protection against PEDV infection.

## 5. Conclusions

In summary, we constructed an anti-PEDV oral probiotic vaccine *L. johnsonii-COE* to deliver the protective antigen COE protein of PEDV. We demonstrated that the immunogenicity of the genetically engineered *L. johnsonii-COE* could efficiently activate DCs mediate Th1-type cell immune response and activate anti-PEDV mucosal, humoral, and cellular immune responses. SIgA and IgG antibodies were delivered to the piglets via the colostrum of immunized sows to resist PEDV infection, implying a potential vaccination strategy for PEDV prevention.

## Figures and Tables

**Table 1:** Primer sequences of cytokines, chemokines.

	Primer Sequence (5′-3′)	Accession Number
β-actin	F- GGTGGGTATGGGTCAGAAAG	AF054837
	R- TCCATGTCGTCCCAGTTGGT	
IL10	F- GGAAGACGTAATGCCGAAGG	NM_214041
	R- GGCACTCTTCACCTCCTCCA	
IL12p40	F- TGGACCTCAGACCAGAGCAG	U08317
	R- GCAGGAGTGACTGGCTCAGA	
CCL-20	F- TGCTCCTGGCTGCTTTGATGTC	AJ577084.1
	R- TCATTGGCGAGCTGCTGTGTG	

## Data Availability

Not applicable.

## Supplementary Materials

The following are available online at https://www.mdpi.com/article/10.3390/v14010007/s1, Table S1: primer sequences of RT-qPCR; and the raw experimental data.
