# Supplementary material for: Immune Responses in Pregnant Sows Induced by Recombinant Lactobacillus johnsonii Expressing the COE Protein of Porcine Epidemic Diarrhea Virus Provide Protection for Piglets against PEDV Infection
Source: Viruses. 2021 Dec 21;14(1):7. doi: 10.3390/v14010007 (PMC8779658; doi:10.3390/v14010007)
Supplement: Supplementary file 1 [file viruses-14-00007-s001.zip › viruses-1394501-supplementary.pdf]

Table S1. Primer sequences of RT-qPCR.

Gene	Primer sequence (5'-3')	Product size (bp)
PEDV-N	F-GGTATTGGAGAAAATCCTGACAGGCATAAGCAACAGCA<br>R- GACGCATCAACACCTTTTTCGACAAATTCCGCATC	251
